# Combined lung and brain ultrasonography for an individualized “brain-protective ventilation strategy” in neurocritical care patients with challenging ventilation needs

**DOI:** 10.1186/s13089-018-0105-4

**Published:** 2018-09-17

**Authors:** Francesco Corradi, Chiara Robba, Guido Tavazzi, Gabriele Via

**Affiliations:** 1 Servizio di Anestesia e Rianimazione, Ente ospedaliero Ospedali Galliera, Genoa, Italy; 2 Anaesthesia and Intensive Care, IRCSS S. Martino Hospital, Genoa, Italy; 3 Neurocritical Care Unit, Addenbrookes Hospital Cambridge, Cambridge, UK; 4 Emergency Department, Anaesthesia and Intensive Care Unit, Fondazione IRCCS Policlinico S. Matteo, Pavia, Italy; 5 Department of Clinical, Surgical, Diagnostic and Paediatric Sciences, Anaesthesia, Intensive Care and Pain Therapy Unit, University of Pavia, Pavia, Italy; 6 Cardiac Anesthesia and Intensive Care, Cardiocentro Ticino, Via Tesserete, 48, Lugano, Switzerland

**Keywords:** ARDS, Brain injury, Lung ultrasound, Brain ultrasound, Neuro-critical care, Respiratory monitoring, Intracranial hypertension, Mechanical ventilation

## Abstract

When intracranial hypertension and severe lung damage coexist in the same clinical scenario, their management poses a difficult challenge, especially as concerns mechanical ventilation management. The needs of combined lung and brain protection from secondary damage may conflict, as ventilation strategies commonly used in patients with ARDS are potentially associated with an increased risk of intracranial hypertension. In particular, the use of positive end-expiratory pressure, recruitment maneuvers, prone positioning, and protective lung ventilation can have undesirable effects on cerebral physiology: they may positively or negatively affect intracranial pressure, based on the final repercussions on PaO_2_ and cerebral perfusion pressure (through changes in cardiac output, mean arterial pressure, venous return, PaO_2_ and PaCO_2_), also according to the baseline conditions of cerebral autoregulation. Lung ultrasound (LUS) and brain ultrasound (BUS, as a combination of optic nerve sheath diameter assessment and cerebrovascular Doppler ultrasound) have independently proven their potential in respectively monitoring lung aeration and brain physiology at the bedside. In this narrative review, we describe how the combined use of LUS and BUS on neurocritical patients with demanding mechanical ventilation needs can contribute to ventilation management, with the aim of a tailored “brain-protective ventilation strategy.”

## Brain injury and severe lung disease: a challenging combination with conflicting therapeutical needs

**Fig. 1:**
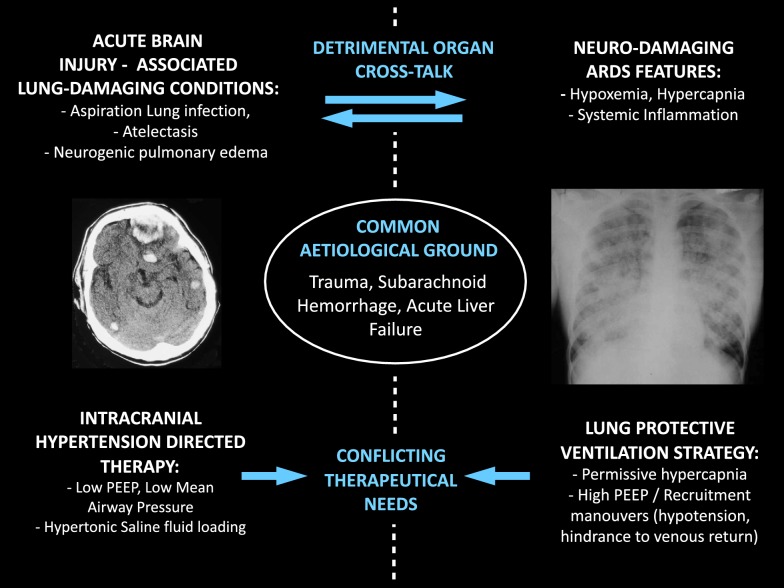
Potential pathophysiological interactions between severe brain injury and severe lung injury, and their conflicting therapeutical needs. Severe brain injury and severe lung damage may ensue as consequence of the same noxious agent (for example in severe multiple trauma or severe liver failure), but also one be the cause of the other (e.g., neurogenic pulmonary edema in subarachnoid hemorrhage, or lung aspiration and infection in a comatous patient). Indeed, once coexisting, disease of one organ can negatively affect the other, in a harmful organ cross-talk (e.g., hypoxemia can worsen brain damage). Finally, some therapeutical interventions directed at protecting one organ may have detrimental effects on the other (e.g., mechanical ventilation strategies can either reduce systemic mean arterial pressure, decrease cerebral venous return, or cause cerebral vasodilation, thus inducing a worsening of intracranial hypertension and reducing the cerebral perfusion pressure)

Intracranial hypertension and severe lung damage may coexist in a variety of clinical settings and pose a difficult challenge, especially in mechanical ventilation management. The two conditions may be caused by the same pathological insult, one be the cause of the other, negatively affect each other in several ways, and in any case may present conflicting therapeutical needs (Fig. 1). This occurs mainly in the context of multiple trauma with severe traumatic brain injury (TBI) [1], but also in patients with subarachnoid hemorrhage [2] and acute liver failure [3]. Acute respiratory syndrome (ARDS) represents altogether a common in-hospital complication after admission for TBI, reaching more than 20% in the adult population [1]. Many ventilation strategies commonly used in patients with ARDS are potentially associated with an increased risk of intracranial hypertension. In particular, the use of positive end-expiratory pressure (PEEP), recruitment maneuvers (RM) and prone positioning (PP) can have undesirable effects on cerebral physiology, by impeding cerebral venous return and decreasing mean arterial pressure (MAP); furthermore, lung protective ventilation (aiming at airway plateau pressures < 28–30 cmH_2_O, driving pressures < 14 cmH_2_O or delta transpulmonary pressures < 10–12 cmH_2_O) may cause hypercarbia, that may result in intracranial pressure (ICP) increases by aggravating a preexisting alteration of cerebral autoregulation [4]. These ARDS ventilation strategies therefore carry a high potential risk for iatrogenic secondary brain damage [5, 6]. Other clinical conditions, even without reaching the severity of ARDS, may pose cerebral protection in conflict with the need of moderate-high PEEP levels. Neurogenic pulmonary edema is a well-known complication of subarachnoid hemorrhage [7], that may represent such a circumstance. Even ventilating an obese neurocritical patient will inevitably raise the conflict between the need to keep the lung open with higher-than-usual PEEP levels [8] and to minimize any hindrance to cerebral venous return. Continuous positive airway pressure has in fact shown to carry the risk of decreasing cerebral perfusion pressure (CPP) and cerebral blood flow (CBF) in both healthy volunteers [9] and brain-injured patients [10].

However, existing data on this phenomenon are conflicting, and must be interpreted with caution. In a first study [9], the drop in CPP and CBF was due to hypocapnic vasoconstriction and not directly related to PEEP. Moreover, the PEEP applied was higher than commonly used in clinical practice and not titrated according to respiratory mechanics. Finally, population studied was composed of spontaneously breathing patients, and the results cannot be assumed to be applicable to deeply sedated patients on invasive mechanical ventilation. In another study [10], alterations of CPP were secondary to MAP reduction and not directly related to PEEP application, but lack of PEEP titration makes the interpretation of data difficult.

Indeed, while properly titrated PEEP can improve cardiac output, especially when left ventricular failure coexists with brain injury, excessive PEEP exposes to lung over-distention and right ventricular impairment [11]. Some authors demonstrated that if PEEP values are below ICP values, then the associated augmentation of intrathoracic pressure does not result in increased ICP [12]. Also, when increased PEEP is applied to brain-injured patients with ARDS, there is a substantial difference in the effects on ICP, depending on whether the application of PEEP causes alveolar hyperinflation or alveolar recruitment and therefore on lung stiffness [13]. Consequently, it is currently unknown which is the optimal level of PEEP in acutely brain-injured patients [14]. Similarly, prone positioning and recruitment maneuvers can increase ICP [6] and their use in brain-injured patients must be weighed in light of the beneficial improvements in oxygenation and gas exchange, which could by far exceed the negative effect of increases of ICP.

In essence, whatever ventilatory adjustment/manouvre may positively or negatively affect ICP in the neurocritical patient, based on its final repercussions on PaO_2_ and on CPP, according to the baseline conditions of cerebral autoregulation.

Recent advances in critical care ultrasound have shown the wide use and clinical impact of point-of-care ultrasonography in critical care units [15] as well as the utility of a multimodal, integrated approach [16].

## Lung ultrasound (LUS) monitoring in the mechanically ventilated patient

International evidence-based consensus on point-of-care lung ultrasound (LUS) [17] recommends its use to track changes in aeration of lung parenchyma, by providing a semi-quantification [18, 19] of these changes and hence monitor lung disease evolution. Several are its applications. LUS can easily *characterize morphologic features of ARDS* [20], *discriminate between focal and diffuse ARDS*, and provide a picture of the heterogeneity of aeration distribution [21]: focal ARDS is characterized by a normal LUS pattern in upper anterior and lateral lung regions and consolidation or B-lines in lower posterior and lateral ones (dependent lung regions in supine position); in diffuse ARDS, which is present in a minority of patients, aeration loss is homogeneously distributed among lung regions, with a diffuse ubiquitous LUS B-pattern. Overdistention cannot be directly measured by LUS and this represents its greatest limitation, although a reduction of physiological lung sliding in anterior regions may be observed when airway pressure is too high [22].

A simple qualitative LUS evaluation has the potential to predict the occurrence of ARDS in blunt trauma patients [23], and *predict the response to PEEP*-*induced lung recruitment* and PEEP-induced increase in PaO_2_ in ARDS patients [24]. Although the same LUS global reareation score applied to ARDS patients failed to predict the response to pronation in terms of oxygenation improvement, basal ARDS morphology described by means of LUS before pronation (focal vs. diffuse morphology) was predictive of effective reareation of dorsal areas, of immediate PaO_2_/FiO_2_ improvement and pCO_2_ decrease [25, 26]. LUS has also proven utility in *discerning different degrees of disease severity at a very early stage of ARDS* [27]. Finally, it has been demonstrated to accurately *track changes in EVLW*, through a bedside quantitative approach [17, 19, 28, 29].

## Brain ultrasonography (BUS) as bedside monitor of cerebral physiology

Brain ultrasonography, including transcranial Doppler (TCD) and the measurement of optic nerve sheath diameter (ONSD), is an evolving technique that has shown promising results in adult [30, 31] and pediatric populations [32] for the non-invasive assessment of ICP (nICP), cerebral perfusion pressure (CPP), as well as for the calculation of advanced parameters such as critical closing pressure and cerebral compliance [33]. Although the invasive intracranial catheter remains the gold standard for ICP measurement, these methods could be helpful when invasive tools are not indicated (i.e., milder than severe degrees of traumatic brain injury) [34], contraindicated (patients with hemostatic disorders) or unavailable (such as in general intensive care) [30].

Increased intrathoracic pressure and consequent ICP elevation produces specific changes in *cerebral arterial blood flow velocity* (FV) waveform that can be assessed by *decreases in the diastolic FV* and *increases in the pulsatility index* (PI = systolic FV − diastolic FV)/mean FV). In mild to moderate TBI, values of PI > 1.25 or a diastolic FV < 25 cm/s are considered pathological, and associated to secondary neurological deterioration [34, 35]. *Increases of ONSD* above a cut-off of 6 mm strongly suggest an increased ICP > 20 mmHg [36, 37]. Venous TCD provides further information. A *reduction of venous FV at the level of the straight sinus* (with a cut-off of 38.50 cm/s) has proven to be a good predictor of ICP above 20 mmHg [36]. Therefore, it is intuitive how this index could be useful in neurocritical care patients with challenging mechanical ventilation conditions, where an important pathophysiological mechanism for ICP increase is related to venous return impairment caused by increased intrathoracic pressures.

In a recent study, *TCD and ONSD were tested as surrogate non*-*invasive measures of ICP changes during prone position and/or PEEP* in non-brain-injured patients undergoing spine surgery [38]: mean ONSD increased significantly upon prone positioning and upon a PEEP application of 8 cmH_2_O, suggesting that these methods may be applicable in the clinical practice to non-invasively monitor cerebrovascular changes during prone position and PEEP increase during mechanical ventilation. ONSD measurement in the prone position can be easily performed by one operator while an assistant holds the head rotated 30 degrees rightward (to measure the right ONSD) and then 30 degrees leftward (to measure the left ONSD) [38].

**Fig. 2:**
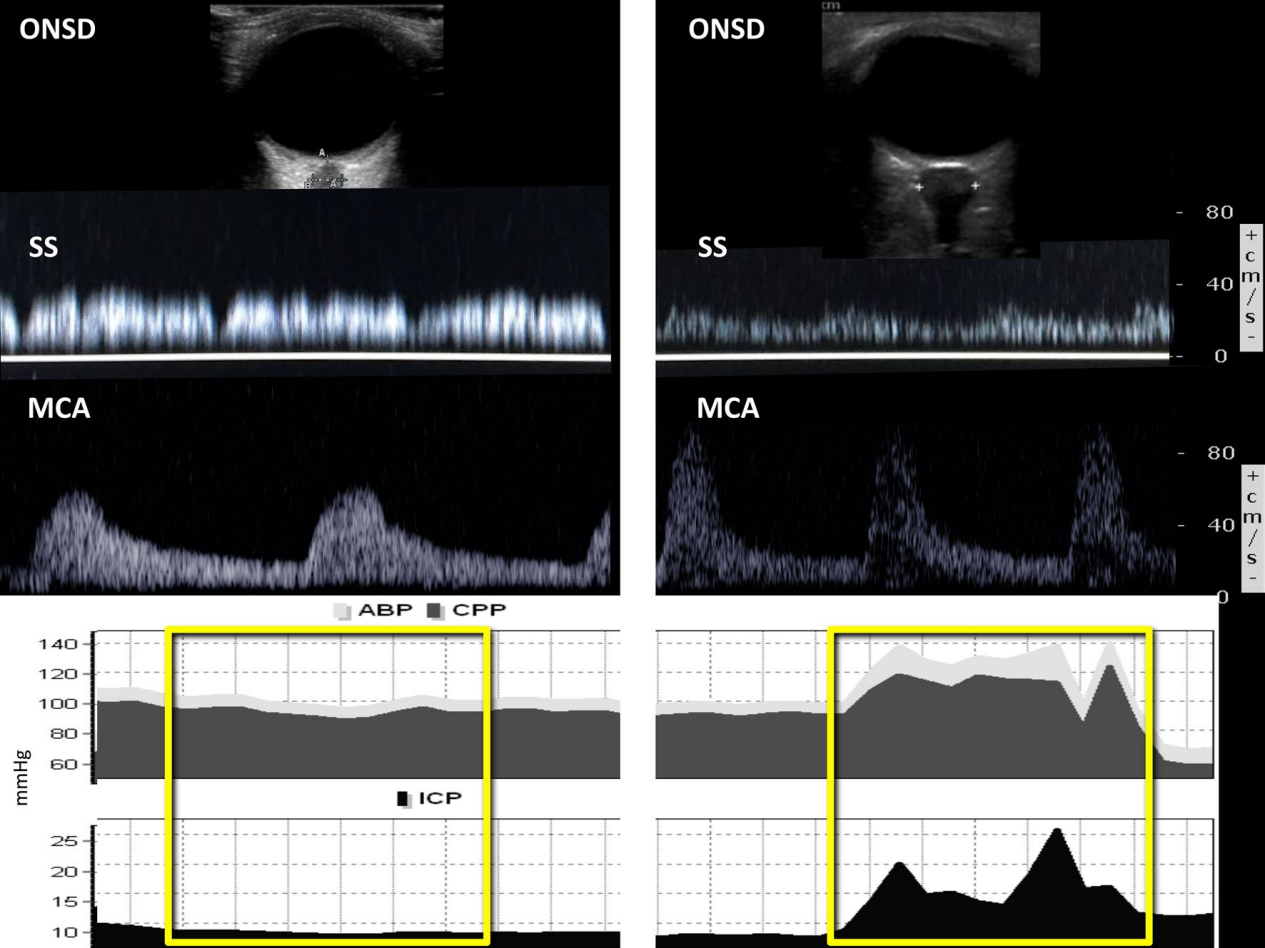
Invasive and non-invasive intracranial pressure monitoring, respectively, through ICP Bolt and through brain ultrasound (BUS, optic nerve sheath diameter and cerebrovascular Doppler sampling) in a patient with traumatic brain injury and ARDS. A 26-year-old lady was admitted to the intensive care unit after a road traffic accident. She presented with severe traumatic brain injury (TBI) and chest trauma with lung contusions. She was monitored with invasive ICP, intubated and mechanically ventilated. On day 2, she developed hypoxic respiratory failure with bilateral atelectasia. We therefore performed recruitment maneuvers with ABP, CPP, and ICP monitoring and concomitant ONSD and TCD measurements (venous TCD on the SS and arterious TCD on the MCA). Recordings and scanning were performed at baseline (left panels) and during a recruitment maneuver and subsequent increase in PEEP level (right panels). Initially, ICP was below 20 mmHg (mean ONSD = 5.2), with PEEP = 8, with stable arterial blood pressure (ABP) and cerebral perfusion pressure (CPP). After recruitment maneuvers and setting PEEP at 16, ICP spiked up > 20 mmHg, with reflex mean systemic arterial blood pressure (ABP) and cerebral perfusion pressure (CPP) increase. BUS showed consistent increase in middle cerebral artery (MCA) pulsatility index (decrease in diastolic flow, increase in systolic flow), reduction in straight sinus (SS) flow, and increase in optic nerve sheath diameter (ONSD = 7 mm). PaCO_2_ remained constant during the procedure and the patients experienced no hypotension nor cardiac output decrease. We therefore reduced PEEP levels and noticed that ICP immediately decreased as well as PI, ONSD and the venous flow on the straight sinus. *MCA* middle cerebral artery, *SS* straight sinus, *ONSD* optic nerve sheath diameter, *ABP* mean systemic arterial blood pressure, *ICP* intracranial pressure, *CPP* cerebral perfusion pressure

Similarly, *BUS can detect relevant changes in cerebrovascular dynamics associated to ICP increases upon recruitment maneuvers* (Fig. 2). In particular, transcranial Doppler has a primarily role in the evaluation of alteration of cerebral blood flow associated with changes in intracranial pressure. However, although data about venous TCD and ONSD are limited in this group of patients, we believe that the use of these complementary techniques should be included in a thorough multimodal brain investigation, but not substitute for it, as they enable to assess cerebral haemodynamics from different perspectives.

Therefore, especially in circumstances when invasive ICP pressure may carry excessive risk, be unavailable or contraindicated, BUS has the potential to provide understanding on whether PEEP, recruitment maneuvers or prone positioning are causing pathological changes in cerebral hemodynamics.

## Ultrasound-guided “brain protective ventilation strategy”

Whenever a patient presents brain injury and requires mechanical ventilation, particular care is recommended in the setting and monitoring of airway pressures and of minute ventilation [39]. The combined use of Lung ultrasonography (LUS) and Brain ultrasonography (BUS) could potentially be used to guide mechanical ventilation and at the same time monitor concurrent relevant cerebrovascular physiology, addressing relevant clinical targets: recruitment maneuvers tolerance, choice of the appropriate PEEP level, minimum tolerable minute ventilation.

**Fig. 3:**
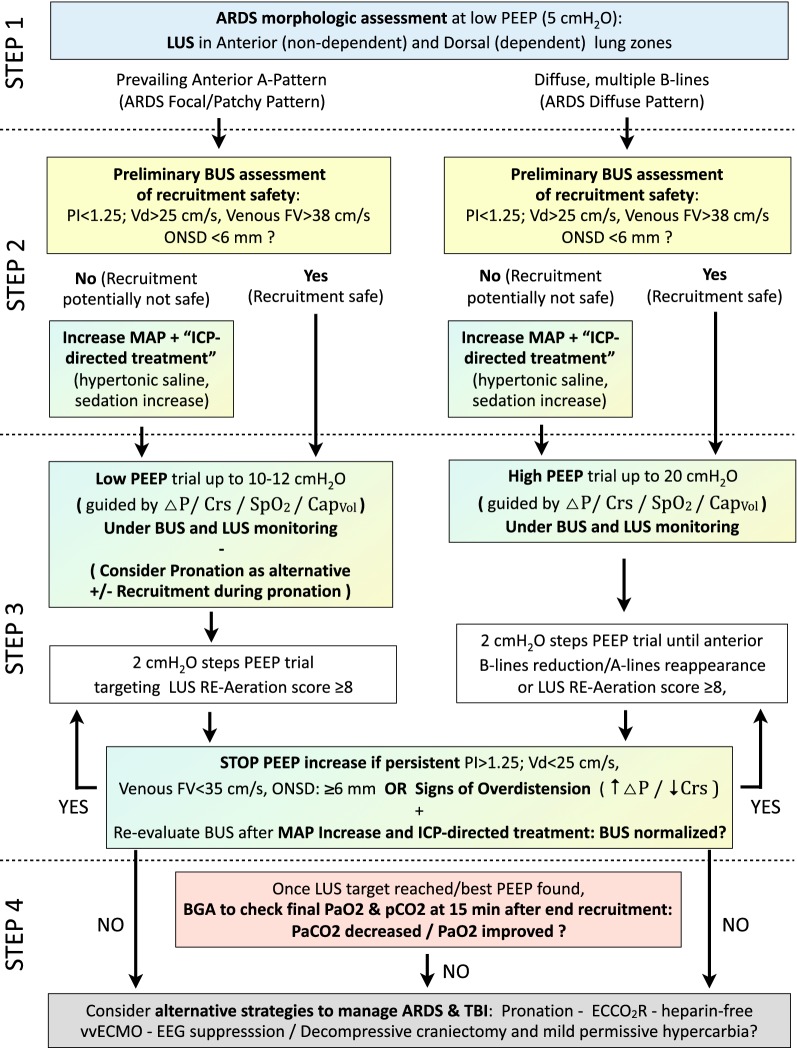
Lung ultrasound–Brain ultrasound (LUS–BUS) combined respiratory and neurological monitoring in patients with traumatic brain injury (TBI) and acute respiratory distress syndrome (ARDS). A four-tiered approach is suggested, in order to decide the best ventilatory strategy and simultaneously monitor the effects on intracranial pressure (ICP) and on cerebrovascular dynamics. The goal is to set the ventilation consistently with a lung-protective strategy without negatively affecting the injured brain. Step 1—scanning of ventral and dorsal chest areas allows to differentiate ARDS with focal/patchy morphology (with less recruitment potential and greater risk of anterior lung overdistention) from ARDS with diffuse, more homogenous, morphology (amenable to successful recruitment at higher PEEP levels). Step 2—once this has been established, the kind of recruitment maneuver suitable for the detected ARDS morphology is preceded by BUS. The detection of signs of intracranial hypertension allows the preemptive institution of medical ICP-directed treatment to reduce the negative impact of the ventilatory maneuvers on the brain. Step 3—the recruiting maneuver is performed [under the guide of driving pressure (Δ*P*) and static respiratory system compliance (*C*_RS_), Volumetric Capnometry, SpO_2_] while monitoring changes in lung aeration (LUS) and signs of their potential negative impact on ICP and cerebrovascular dynamics and (BUS). Step 4—the final effect of the recruitment maneuver and the chosen PEEP is finally assessed, both in terms of gas exchanges, lung mechanics, and of net effect on the ICP and cerebrovascular dynamics. Should the ventilation target not be reachable nor compatible with brain protection, other respiratory support strategies/ICP treatments should be considered. *ARDS* acute respiratory distress syndrome, *LUS* lung ultrasound, *BUS* brain ultrasound, *PEEP* positive end expiratory pressure, *PI* middle cerebral artery pulsatility index, *V*_*d*_ middle cerebral artery diastolic arterial flow velocity, *FV* flow velocity; *ONSD* optic nerve sheath diameter, *MAP* mean systemic arterial pressure, *ICP* intracranial pressure, *BGA* blood gas analysis, *TBI* traumatic brain injury, *ECCOR* extracorporeal CO_2_ removal, *vvECMO* veno-venous extracorporeal membrane oxygenation, *EEG* electroencephalography, *C*_*RS*_ respiratory system compliance, *ΔP* driving pressure, *Cap*_*Vol*_ volumetric capnometry, *SpO*_*2*_ arterial oxygen saturation

To tackle the challenging issue of *combined lung and brain protection from secondary damage* in the brain-injured patient under demanding mechanical ventilation, a pragmatic approach to orient mechanical ventilation settings based on LUS and BUS can be proposed, as part of the multimodal, multi-organ monitoring of such complex patients. This approach is based on the concept of noninvasively screening for the potential of a reduced tolerance to ventilatory maneuvers (BUS signs of high ICP/reduced cerebral flow), and of monitoring the cerebral effect itself of the ventilator maneuvers once performed. Where ICP invasive monitoring, that should be considered the gold standard, and other brain monitoring tools are already in place, this approach would represent a complimentary simultaneous assessment of lung recruitability/effective recruitment, screening for the potential of a reduced tolerance to ventilatory maneuvers (BUS signs of high ICP/reduced cerebral flow) and brain response to ventilatory manipulations. When ICP invasive monitoring is not indicated, contraindicated or not available, the combined LUS–BUS approach may represent a non-invasive monitoring tool instrumental to tackle the double challenge of concomitant lung- and neuro-protection in mechanically ventilated patients. A four-tiered LUS–BUS approach to the most severe setting of ARDS in TBI is here presented as example of this concept (Fig. 3).

Essentially, a tailored PEEP-trial is performed under guidance of respiratory mechanics/monitoring parameters [driving pressure (Δ*P*), Respiratory System Compliance, SpO_2_, Volumetric Capnometry], and its effects are then monitored with LUS [40] and BUS [38]. The first step is *the evaluation of lung morphology* at a PEEP of 5 cmH_2_O. This can be done by examination of anterior and dorsal chest areas: the presence of a prevailing normal A pattern [17] or scarce B-lines in the anterior areas, and consolidations in the dorsal ones characterizes focal aeration loss; conversely, if diffuse multiple well-separated or coalescent B-lines (B pattern) [17] are found in anterior areas of the chest, a diffuse, more homogeneous loss of aeration is demonstrated.

The second step focuses on the *screening for BUS signs of intracranial hypertension*: should these be detected (thus making the PEEP-trial more likely to adversely affect brain vascular dynamics), the expected improvement in oxygenation and hypercarbia upon reduction of alveolar dead space may still be worth the transient side effects of the recruiting PEEP-trial (in terms of cerebral venous return reduction and pCO_2_ increase). This should though be performed only after ICP-reducing treatments are delivered (sedation, hypertonic saline boluses).

As third step, the *PEEP trial is performed monitoring respiratory mechanics as reference*. For patients with focal loss of aeration, low levels of PEEP (≤ 10 cmH_2_O) can be tested, under BUS monitoring to promptly detect cerebral hemodynamics disturbances (higher levels of PEEP rarely result in recruitment of consolidations in these patients, and expose normally aerated lung zones to over-distension, with no benefit on gas exchanges at the useless price of increasing ICP). For patients with diffuse loss of aeration (diffuse ARDS), higher levels of PEEP (from 12 cmH_2_O up) can be tested, still under BUS monitoring. At each increase of PEEP, responsiveness can be monitored by LUS examination of anterior zones [40]; PEEP is increased by 2–4 cmH_2_O until anterior zones with moderate or severe aeration loss (respectively separated and coalescent B-lines) become normal (A-lines). Increasing PEEP further to obtain a complete disappearance of B-lines (in lateral zones) or consolidation (in posterior zones) theoretically exposes previously recruited anterior lung zones to over-distension. A semi-quantitative re-aeration score can also be applied in a more exhaustive approach: each intercostal space is examined within each of the 12 regions in both hemi-thoraxes [17]. To apply this scoring method, the most pathological pattern in each region is considered as characterizing the pattern of the entire region. A LUS score of re-aeration would be then calculated, based on the changes of the ultrasound pattern in each region of interest from baseline to end to the recruiting PEEP-trial (a re-aeration score of ≥ 8 has been associated to a CT-scan measured recruitment greater than 600 ml) [24]. The appearance of BUS signs of worsening intracranial hypertension or respiratory mechanics signs of overdistension warrants cessation of the maneuver and ICP-directed medical treatment.

The final step consists in the *evaluation of the final result of the PEEP*-*trial*. At the chosen PEEP level and after sufficient time for gas exchanges to stabilize, BGA assessment will describe the PaO_2_ and PaCO_2_ obtained. Should the new ventilation setting yield no improvement in arterial blood gases, or should this improvement have occurred at the expenses of excessive ICP (persistent BUS signs of elevated ICP), alternative respiratory support strategies [41, 42] or TBI treatments [43] could be considered.

## Conclusion

Respiratory and cerebral monitoring are complex parts of critical care. We herewith presented the concept of a combined bedside ultrasound respiratory and neurological monitoring of the neurocritical patient on mechanical ventilation, based on recent evidence suggesting the added value that both techniques represent for bedside monitoring. At present, the use of PEEP to treat ARDS in TBI patients may be appropriate; but the decision to increase PEEP in a neurocritical patient should be accompanied by maintenance of systemic arterial pressure and gas-exchange stability, and a close monitoring of cerebral parameters (mainly ICP and CPP). The concomitant combined use of ultrasound for lung and brain monitoring in the challenging setting of co-existing TBI and ARDS could serve the purpose of guiding the ventilator strategy (PEEP setting, recruitment vs. pronation) and of promptly screening for signs of increased/worsening intracranial hypertension (i.e., screening for tolerance to PEEP, to sub-optimal PaCO_2_). However, LUS–BUS monitoring should be integrated into the well established diagnostic-monitoring workup, including the evaluation of respiratory mechanics, gas-exchange, computer tomography scan and multi-modal brain monitoring. Further research is advocated to validate this ultrasound-aided approach.

## References

[1] Rincon F, Ghosh S, Dey S, Maltenfort M, Vibbert M, Urtecho J, McBride W, Moussouttas M, Bell R, Ratliff JK, Jallo J (2012). Impact of acute lung injury and acute respiratory distress syndrome after traumatic brain injury in the United States. Neurosurgery.

[2] Veeravagu A, Chen YR, Ludwig C, Rincon F, Maltenfort M, Jallo J, Choudhri O, Steinberg GK, Ratliff JK (2014). Acute lung injury in patients with subarachnoid hemorrhage: a nationwide inpatient sample study. World Neurosurg.

[3] Damm TW, Kramer DJ (2016). The liver in critical illness. Crit Care Clin.

[4] Meng L, Gelb AW (2015). Regulation of cerebral autoregulation by carbon dioxide. Anesthesiology.

[5] Beuret P, Carton MJ, Nourdine K, Kaaki M, Tramoni G, Ducreux JC (2002). Prone position as prevention of lung injury in comatose patients: a prospective, randomized, controlled study. Intensive Care Med.

[6] Roth C, Ferbert A, Deinsberger W, Kleffmann J, Kastner S, Godau J, Schuler M, Tryba M, Gehling M (2014). Does prone positioning increase intracranial pressure? A retrospective analysis of patients with acute brain injury and acute respiratory failure. Neurocrit Care.

[7] Bruder N, Rabinstein A, Participants in the International Multi-Disciplinary Consensus Conference on the Critical Care Management of Subarachnoid H (2011). Cardiovascular and pulmonary complications of aneurysmal subarachnoid hemorrhage. Neurocrit Care.

[8] Wang C, Zhao N, Wang W, Guo L, Guo L, Chi C, Wang X, Pi X, Cui Y, Li E (2015). Intraoperative mechanical ventilation strategies for obese patients: a systematic review and network meta-analysis. Obes Rev.

[9] Yiallourou TI, Odier C, Heinzer R, Hirt L, Martin BA, Stergiopulos N, Haba-Rubio J (2013). The effect of continuous positive airway pressure on total cerebral blood flow in healthy awake volunteers. Sleep Breath.

[10] Shapiro HM, Marshall LF (1978). Intracranial pressure responses to PEEP in head-injured patients. J Trauma.

[11] Mekontso Dessap A, Boissier F, Charron C, Begot E, Repesse X, Legras A, Brun-Buisson C, Vignon P, Vieillard-Baron A (2016). Acute cor pulmonale during protective ventilation for acute respiratory distress syndrome: prevalence, predictors, and clinical impact. Intensive Care Med.

[12] McGuire G, Crossley D, Richards J, Wong D (1997). Effects of varying levels of positive end-expiratory pressure on intracranial pressure and cerebral perfusion pressure. Crit Care Med.

[13] Mascia L, Grasso S, Fiore T, Bruno F, Berardino M, Ducati A (2005). Cerebro-pulmonary interactions during the application of low levels of positive end-expiratory pressure. Intensive Care Med.

[14] Young N, Rhodes JK, Mascia L, Andrews PJ (2010). Ventilatory strategies for patients with acute brain injury. Curr Opin Crit Care.

[15] Zieleskiewicz L, Muller L, Lakhal K, Meresse Z, Arbelot C, Bertrand PM, Bouhemad B, Cholley B, Demory D, Duperret S, Duranteau J, Guervilly C, Hammad E, Ichai C, Jaber S, Langeron O, Lefrant JY, Mahjoub Y, Maury E, Meaudre E, Michel F, Muller M, Nafati C, Perbet S, Quintard H, Riu B, Vigne C, Chaumoitre K, Antonini F, Allaouchiche B, Martin C, Constantin JM, De Backer D, Leone M, Car’Echo, AzuRea Collaborative N (2015). Point-of-care ultrasound in intensive care units: assessment of 1073 procedures in a multicentric, prospective, observational study. Intensive Care Med.

[16] Bataille B, Riu B, Ferre F, Moussot PE, Mari A, Brunel E, Ruiz J, Mora M, Fourcade O, Genestal M, Silva S (2014). Integrated use of bedside lung ultrasound and echocardiography in acute respiratory failure: a prospective observational study in ICU. Chest.

[17] Volpicelli G, Elbarbary M, Blaivas M, Lichtenstein DA, Mathis G, Kirkpatrick AW, Melniker L, Gargani L, Noble VE, Via G, Dean A, Tsung JW, Soldati G, Copetti R, Bouhemad B, Reissig A, Agricola E, Rouby JJ, Arbelot C, Liteplo A, Sargsyan A, Silva F, Hoppmann R, Breitkreutz R, Seibel A, Neri L, Storti E, Petrovic T, International Liaison Committee on Lung Ultrasound for International Consensus Conference on Lung U (2012). International evidence-based recommendations for point-of-care lung ultrasound. Intensive Care Med.

[18] Corradi F, Ball L, Brusasco C, Riccio AM, Baroffio M, Bovio G, Pelosi P, Brusasco V (2013). Assessment of extravascular lung water by quantitative ultrasound and CT in isolated bovine lung. Respir Physiol Neurobiol.

[19] Corradi F, Brusasco C, Vezzani A, Santori G, Manca T, Ball L, Nicolini F, Gherli T, Brusasco V (2016). Computer-aided quantitative ultrasonography for detection of pulmonary edema in mechanically ventilated cardiac surgery patients. Chest.

[20] Lichtenstein D, Goldstein I, Mourgeon E, Cluzel P, Grenier P, Rouby JJ (2004). Comparative diagnostic performances of auscultation, chest radiography, and lung ultrasonography in acute respiratory distress syndrome. Anesthesiology.

[21] Corradi F, Brusasco C, Pelosi P (2014). Chest ultrasound in acute respiratory distress syndrome. Curr Opin Crit Care.

[22] Markota A, Golub J, Stozer A, Fluher J, Prosen G, Bergauer A, Svensek F, Sinkovic A (2016). Absence of lung sliding is not a reliable sign of pneumothorax in patients with high positive end-expiratory pressure. Am J Emerg Med.

[23] Leblanc D, Bouvet C, Degiovanni F, Nedelcu C, Bouhours G, Rineau E, Ridereau-Zins C, Beydon L, Lasocki S (2014). Early lung ultrasonography predicts the occurrence of acute respiratory distress syndrome in blunt trauma patients. Intensive Care Med.

[24] Bouhemad B, Brisson H, Le-Guen M, Arbelot C, Lu Q, Rouby JJ (2011). Bedside ultrasound assessment of positive end-expiratory pressure-induced lung recruitment. Am J Respir Crit Care Med.

[25] Haddam M, Zieleskiewicz L, Perbet S, Baldovini A, Guervilly C, Arbelot C, Noel A, Vigne C, Hammad E, Antonini F, Lehingue S, Peytel E, Lu Q, Bouhemad B, Golmard JL, Langeron O, Martin C, Muller L, Rouby JJ, Constantin JM, Papazian L, Leone M, Network CAEC, AzuRea Collaborative N (2016). Lung ultrasonography for assessment of oxygenation response to prone position ventilation in ARDS. Intensive Care Med.

[26] Guerin C, Gattinoni L (2016). Assessment of oxygenation response to prone position ventilation in ARDS by lung ultrasonography. Intensive Care Med.

[27] Zhao Z, Jiang L, Xi X, Jiang Q, Zhu B, Wang M, Xing J, Zhang D (2015). Prognostic value of extravascular lung water assessed with lung ultrasound score by chest sonography in patients with acute respiratory distress syndrome. BMC Pulm Med.

[28] Volpicelli G, Skurzak S, Boero E, Carpinteri G, Tengattini M, Stefanone V, Luberto L, Anile A, Cerutti E, Radeschi G, Frascisco MF (2014). Lung ultrasound predicts well extravascular lung water but is of limited usefulness in the prediction of wedge pressure. Anesthesiology.

[29] Agricola E, Bove T, Oppizzi M, Marino G, Zangrillo A, Margonato A, Picano E (2005). “Ultrasound comet-tail images”: a marker of pulmonary edema: a comparative study with wedge pressure and extravascular lung water. Chest.

[30] Moretti R, Pizzi B (2009). Optic nerve ultrasound for detection of intracranial hypertension in intracranial hemorrhage patients: confirmation of previous findings in a different patient population. J Neurosurg Anesthesiol.

[31] Whiteley JR, Taylor J, Henry M, Epperson TI, Hand WR (2015). Detection of elevated intracranial pressure in robot-assisted laparoscopic radical prostatectomy using ultrasonography of optic nerve sheath diameter. J Neurosurg Anesthesiol.

[32] Albin MS (2014). Measuring the ICP in neonates and infants noninvasively is also important. J Neurosurg Anesthesiol.

[33] Robba C, Cardim D, Sekhon M, Budohoski K, Czosnyka M (2018). Transcranial Doppler: a stethoscope for the brain-neurocritical care use. J Neurosci Res.

[34] Bouzat P, Francony G, Declety P, Genty C, Kaddour A, Bessou P, Brun J, Jacquot C, Chabardes S, Bosson JL, Payen JF (2011). Transcranial Doppler to screen on admission patients with mild to moderate traumatic brain injury. Neurosurgery.

[35] Bouzat P, Almeras L, Manhes P, Sanders L, Levrat A, David JS, Cinotti R, Chabanne R, Gloaguen A, Bobbia X, Thoret S, Oujamaa L, Bosson JL, Payen JF, Asehnoune K, Pes P, Lefrant JY, Mirek S, Albasini F, Scrimgeour C, Thouret JM, Chartier F, Ginet M, Investigators T-TS (2016). Transcranial Doppler to predict neurologic outcome after mild to moderate traumatic brain injury. Anesthesiology.

[36] Robba C, Cardim D, Tajsic T, Pietersen J, Bulman M, Donnelly J, Lavinio A, Gupta A, Menon DK, Hutchinson PJA, Czosnyka M (2017). Ultrasound non-invasive measurement of intracranial pressure in neurointensive care: a prospective observational study. PLoS Med.

[37] Ohle R, McIsaac SM, Woo MY, Perry JJ (2015). Sonography of the optic nerve sheath diameter for detection of raised intracranial pressure compared to computed tomography: a systematic review and meta-analysis. J Ultrasound Med.

[38] Robba C, Bragazzi NL, Bertuccio A, Cardim D, Donnelly J, Sekhon M, Lavinio A, Duane D, Burnstein R, Matta B, Bacigaluppi S, Lattuada M, Czosnyka M (2017). Effects of prone position and positive end-expiratory pressure on noninvasive estimators of ICP: a pilot study. J Neurosurg Anesthesiol.

[39] Rajajee V, Riggs B, Seder DB (2017). Emergency neurological life support: airway, ventilation, and sedation. Neurocrit Care.

[40] Bouhemad B, Mongodi S, Via G, Rouquette I (2015). Ultrasound for “lung monitoring” of ventilated patients. Anesthesiology.

[41] Munoz-Bendix C, Beseoglu K, Kram R (2015). Extracorporeal decarboxylation in patients with severe traumatic brain injury and ARDS enables effective control of intracranial pressure. Crit Care.

[42] Wu SC, Chen WT, Lin HH, Fu CY, Wang YC, Lo HC, Cheng HT, Tzeng CW (2015). Use of extracorporeal membrane oxygenation in severe traumatic lung injury with respiratory failure. Am J Emerg Med.

[43] Stocchetti N, Maas AI (2014). Traumatic intracranial hypertension. N Engl J Med.

